# Loneliness during the COVID-19 pandemic: A potential risk factor for post-pandemic cognitive decline in older adults without dementia

**DOI:** 10.1590/1980-5764-DN-2024-0256

**Published:** 2025-06-02

**Authors:** Alaa Harb, Juliana Nery Souza-Talarico, Grace Willey, Fernanda Carini da Silva, Maria Clara Ferreira de Jesus, Jennifer Deberg

**Affiliations:** 1 University of Iowa, College of Nursing, Iowa City, USA.; 2 Univeridade de São Paulo, Escola de Enfermagem, São Paulo SP, Brazil.; 3 University of Iowa, Hardin Library for the Health Sciences, Iowa City, USA.

**Keywords:** COVID-19, Loneliness, Social Isolation, Aging, Cognitive Dysfunction, COVID-19, Solidão, Isolamento Social, Envelhecimento, Disfunção Cognitiva

## Abstract

The COVID-19 pandemic has exacerbated loneliness and social isolation among older adults, raising concerns about their mental and physical well-being. While loneliness’s adverse effects on health are well-documented, its specific impact on cognitive health during the pandemic remains under investigation.

**Objective:**

The aim of this study was to examine the association between loneliness during the COVID-19 pandemic and cognitive decline in cognitively unimpaired older adults by synthesizing evidence from primary studies.

**Methods:**

A comprehensive search strategy was employed across multiple databases, including MEDLINE (PubMed), CINAHL (EBSCO), PsycINFO (EBSCO), EMBASE, Scopus, AgeLine, and ProQuest, following PRISMA guidelines. Studies were selected to examine the association between loneliness and cognitive function in cognitively unimpaired older adults (aged ≥50 years) during the COVID-19 pandemic.

**Results:**

A total of eight studies were included from 1,384 search results, reviewing data from 10,449 cognitively unimpaired older adults. A total of six studies found significant associations between loneliness or social isolation and subjective cognitive decline (SCD). Only one study linked loneliness to lower objective cognitive performance. Notably, half of the studies considered key covariates, such as depression, which could mediate the relationship between loneliness and cognitive decline.

**Conclusion:**

Loneliness during the COVID-19 pandemic is linked to SCD in older adults, though objective evidence is limited. The pandemic underscored the long-term impact of social isolation on cognition and mental health, highlighting the need for standardized neuropsychological tools and key covariates in studies to identify those at risk.

## RESUMO

A pandemia da COVID-19 exacerbou a solidão e o isolamento social entre idosos, levantando preocupações sobre seu bem-estar mental e físico. Embora os efeitos adversos da solidão na saúde sejam bem documentados, seu impacto específico na saúde cognitiva durante a pandemia continua sob investigação.

**Objetivo:**

Examinar a associação entre solidão durante a COVID-19 e declínio cognitivo em adultos de meia idade e idosos cognitivamente saudáveis, sintetizando evidências de estudos primários.

**Métodos:**

Uma estratégia de busca abrangente foi empregada em vários bancos de dados, incluindo MEDLINE (PubMed), CINAHL (EBSCO), PsycINFO (EBSCO), EMBASE, Scopus, AgeLine e ProQuest, seguindo as diretrizes PRISMA. Estudos foram selecionados para examinar a associação entre solidão e função cognitiva em adultos mais velhos e idosos cognitivamente saudaveis (com idade ≥50 anos) durante a pandemia da COVID-19.

**Resultados:**

Oito estudos foram incluídos de 1.384 resultados de pesquisa, revisando dados de 10.449 adultos mais velhos cognitivamente não prejudicados. Seis estudos encontraram associações significativas entre solidão ou isolamento social e declínio cognitivo subjetivo. Apenas um estudo vinculou a solidão a um desempenho cognitivo objetivo mais baixo. Notavelmente, metade dos estudos considerou covariáveis-chave, como depressão, que poderiam mediar a relação entre solidão e declínio cognitivo.

**Conclusão:**

A solidão durante a COVID-19 está ligada ao declínio cognitivo subjetivo em adultos mais velhos, embora as evidências objetivas sejam limitadas. A pandemia ressaltou o impacto de longo prazo do isolamento social na cognição e na saúde mental, destacando a necessidade de ferramentas neuropsicológicas padronizadas e covariáveis-chave em estudos para identificar aqueles em risco.

## INTRODUCTION

Loneliness during aging represents a profound and prevalent challenge, particularly related to a series of pivotal life transitions, such as retirement, the demise of contemporaries, and health decline that restricts physical mobility, thus severing established social ties even among those without pre-existing cognitive impairments^1^. Loneliness is characterized by an individual’s perceived deficiency in meaningful social connections and companionship and can emerge as a potential maladaptive response to the exigencies of social isolation^1,2^. For older adults, the consequences of loneliness are far-reaching, manifesting in increased susceptibility to a range of health complications, including but not limited to cardiovascular issues, neurological abnormalities, and compromised immune function. The attenuated social interaction impairs older adults’ perceived wellness and quality of life^3,4^.

Pre-pandemic research established a link between social isolation risk factors, such as solitary living and limited social networks, and an elevated risk of Alzheimer’s disease and related dementias (ADRDs) among older adults^5,6^. The advent of the COVID-19 pandemic has significantly magnified the prevalence of loneliness, affecting diverse population groups, with older adults bearing a disproportionate burden^3^. This group’s inherent dependency on social support systems, coupled with their heightened vulnerability to the virulence and mortality risks associated with the COVID-19 pandemic, has spotlighted the exacerbated challenges and experiences with deteriorations in well-being, cognitive functionality, and increased anxiety levels^7^.

Two recent meta-analyses have highlighted the prevalence of loneliness and social isolation during the COVID-19 pandemic, focusing on older adults. The first meta-analysis found a significant increase in loneliness (28.6%) and social isolation (31.2%) among older adults during the pandemic^8^. Notably, the prevalence was higher in studies conducted more than 3 months after the pandemic began, indicating the worsening effects of prolonged isolation. This study included both cognitively healthy individuals and those with cognitive impairments, focusing mainly on community-dwelling older adults^8^.

The second meta-analysis specifically examined the impact of social isolation on cognitive function and mental health among older adults, including those with dementia. It found that older adults with dementia were particularly vulnerable, showing a higher prevalence of cognitive decline and behavioral and psychological symptoms of dementia (BPSD). The proportion of cognitively impaired individuals experiencing worsening symptoms was nearly double that of healthy older adults facing subjective cognitive decline (SCD)^9^. This highlights that patients with existing cognitive disorders are more vulnerable to further cognitive decline when facing social isolation, which might impact the generalization of findings to cognitively unimpaired older adults.

The pandemic’s unique social disruptions raise critical inquiries about its impact on cognitive decline or dementia progression, especially among those without prior cognitive impairments^9,10^. The planned review will focus specifically on cognitively unimpaired older adults living in the community, aiming to explore how social isolation and loneliness contribute to cognitive decline in this group. Given that previous studies have focused on individuals with pre-existing cognitive impairments, this new review will address a gap in the literature and provide insights into how these factors affect those without cognitive disorders.

Understanding the relationship between loneliness and cognitive decline during the pandemic has become an essential area of research, as it holds potential implications for long-term consequences, significantly increasing the risk for ADRDs^9^. This review aimed to examine the association between loneliness during the COVID-19 pandemic and cognitive decline in cognitively unimpaired older adults living in their communities.

## METHODS

**Figure 1:**
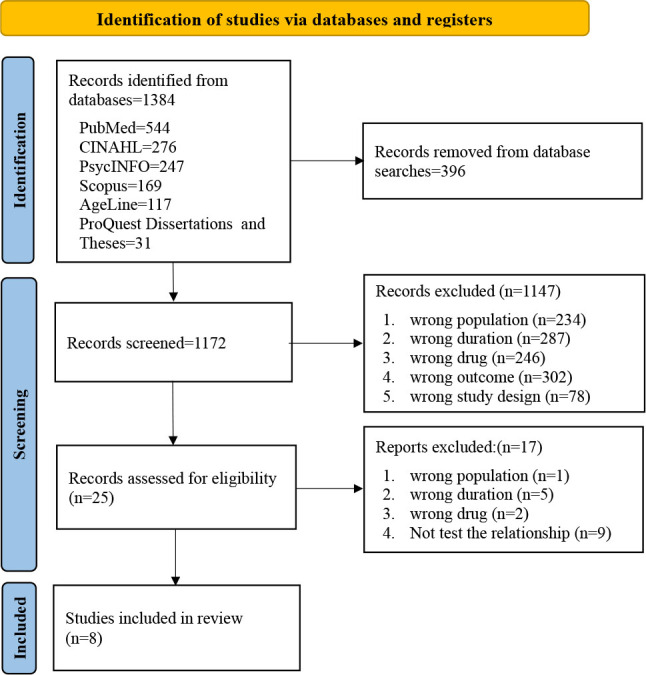
PRISMA flow diagram of the study selection process.

This review followed the JBI guidelines for systematic reviews^11^. These guidelines informed the inclusion criteria, search strategy, selection process, assessment of methodological quality, and data extraction, items, and synthesis (Figure 1). The Population, Exposure of Interest, and Outcome (PEO) framework was used to determine whether studies were eligible for inclusion.

### Inclusion criteria

#### Population

Studies that included non-institutionalized, independently living older adults aged 50 years and older without a diagnosis of cognitive impairment or dementia determined.

#### Exposure of interest

Studies were included if data collection occurred at least during the COVID-19 pandemic (from March 11, 2020, to May 5, 2023)^12^. Social measures to mitigate the COVID-19 spread (i.e., social distancing or social isolation/lockdown) were examined.

#### Outcomes

Only studies that examined the relationship between loneliness related to the COVID-19 pandemic and cognitive decline were included. Studies should have assessed loneliness using valid scales or self-reports and measured cognitive performance through global cognition, attention, short- and long-term memory, verbal fluency, and executive function tests.

#### Types of studies

Experimental or quasi-experimental designs and observational studies include prospective or retrospective cohort studies, case–control studies, and cross-sectional studies.

### Three-step search strategy

#### First step

An initial limited search of MEDLINE (PubMed), CINAHL (EBSCO), PsycINFO (EBSCO), Scopus, and AgeLine was undertaken, followed by an analysis of the text words in the titles, abstracts, and index terms to test the adequacy of the terms and strategies. Search strategies are presented in Supplementary Material (available at https://www.demneuropsy.com.br/wp-content/uploads/2025/02/DN-2024.0256-Supplementary-Material.docx).

#### Second step

A second comprehensive search of MEDLINE (PubMed), CINAHL (EBSCO), EMBASE, PSYCHNFO (EBSCO), Scopus, and ProQuest was conducted using all keywords and index terms identified in the previous step. The search strategies for each database are shown in Supplementary Material.

#### Third step

After the two electronic searches, a hand search of all the reference lists of the articles selected for the critical appraisal was conducted to identify any potentially eligible studies not uncovered by the earlier searches. An expert librarian (JD) managed all literature database searches.

### Selection process

Studies were first imported into EndNote to remove duplicates. Following the inclusion criteria, researchers (GW, AH, MCJ, and FS) independently reviewed all titles and abstracts from the retrieved records and reviewed and discussed full-text versions of the studies for inclusion. A third reviewer (JNST) consulted with and arbitrated any within-pair disagreements.

### Assessment of methodological quality

The methodological quality of all selected papers was appraised using the JBI Checklist for Analytical Cross-Sectional Studies, which includes eight questions. Studies scoring ≥0.8 on a 1.0-point scale were considered of high quality. Studies were tagged as “potential risk for bias” if the exposure (COVID-19 pandemic) and outcomes (cognitive function and loneliness) were not adequately detailed^13^. The quality appraisal also accounted for missing information on controlling potential confounders in regression models like multiple logistic regression and Cox proportional hazard regression. The researchers conducted independent critical appraisals, discussed their findings, and resolved disagreements with a third reviewer.

### Data items and extraction

Data items included study information, methods, participant characteristics, outcome measurements, and study results (Table 1). Data from eligible studies were extracted using the JBI SUMARI data extraction template (https://sumari.jbi.global/features). Reviewers first extracted data independently and then compared their results. Discrepancies were resolved through discussion with a third reviewer.

**Table 1:** Fluids and biomarkers in Alzheimer’s disease.

Extraction topic	Items extracted
Study information and methods	Author, year of publication, study design, country, and setting
Participant characteristics	Age, sex, education, race, ethnicity, and sample size
Measurements	Social distancing measures: voluntary “stay-at-home” and enforced “stay-at-home” (i.e., lockdown) Loneliness: scale or questionnaire name Social isolation: scale or questionnaire name Cognitive performance: cognitive or self-complaint questionnaire name
Study results	Correlation coefficients, mean difference, odds ratio and, respectively, confidence intervals between loneliness and cognitive performance and crude and adjusted models

### Data synthesis

Effect sizes, expressed as odds ratios (for categorical data), weighted mean differences (for continuous data), and 95% confidence intervals were used to analyze the association between the COVID-19 pandemic and cognitive function in older adults. The findings were presented in narrative form and a tabular summary to aid interpretation.

## RESULTS

### Studies and sample characteristics

Eight peer-reviewed manuscripts were selected from an initial pool of 1,384 studies, comprising six longitudinal and two cross-sectional findings from seven countries. The total sample size included 10,449 participants, predominantly female and cognitively unimpaired older adults. Most of the studies focused on participants who adhered to voluntary stay-at-home requests as a government measure to mitigate the spread of the virus (Table 2)^14-21^.

**Table 2:** The citation, study design, country, sample size, participant age, gender, and race, social distancing measures, loneliness, social isolation, and cognition measures and outcomes for each included study.

Author	Study design/country	No. of female (%)	Population			Loneliness or social isolation	Measures		Outcomes
			Mean age (range)	Race (%)	Social distancing measures		Cognition	Covariates	Loneliness or social isolation × cognition
Noguchi et al.^14^	Longitudinal/Japan	955 (54.7)	79.8 (NA)	NA	Voluntary stay-at-home	Social isolation index	Subjective cognitive decline	Age, BADL	Social isolation was associated with cognitive decline
Okely et al.^15^	Longitudinal/Scotland	137 (48.2)	84.0 (NA)	NA	Enforced stay-at-home (lockdown)	Loneliness 1-item	Subjective memory decline	Age, sex, education, household composition, DM, CVD, anxiety, depression, intellect, emotional stability, personality	No significant association
Kobayashi et al.^16^	Longitudinal/US	2,262 (58.2)	68.2 (NA)	W (84.3)	Mixed or voluntary and enforced stay-at-home across US states	3-item UCLA Loneliness Scale	Subjective cognitive decline	Age, race, education, relationship, employment, previous diagnosis, mobility aid, social isolation, depressive symptoms	The higher the loneliness scores, the lower the cognitive function
Li et al.^17^	Cross-sectional /Japan	706* (44.6)	NA (70–89)	NA	Voluntary stay-at-home	3-item UCLA Loneliness Scale	Subjective cognitive decline	Sex, SES, education, physical activity, smoking, comorbidities, depression, COVID-19 anxiety, ICT	The higher the loneliness scores, the higher the subjective cognitive decline
Nogueira et al.^18^	Longitudinal/Portugal	150 (74.4)	69.0 (51–91)	NA	Enforced stay-at-home (lockdown)	16-item UCLA Loneliness Scale	Subjective cognitive decline MMSE, MoCA, Trail Making Test, Digital Symbol Coding, Digit Span, and verbal fluency	NA	The higher the loneliness scores, the higher the subjective cognitive decline No association between loneliness and cognitive performance on tests
Durón-Reyes et al.^19^	Cross-sectional/Mexico	305 (75.7)	65.4 (NA)	NA	Voluntary stay-at-home	De Jong Gierveld Loneliness Scale	Subjective cognitive decline	Age, sex, and education	The higher the loneliness scores, the higher the subjective cognitive decline
Corbett et al.^20^	Longitudinal/UK	3,142 (54)	67.5 (50–96)	W (98.7) B (0.1) O (0.2) A (0.7) NR (0.6)	Enforced stay-at-home (lockdown)	Loneliness 2-item	Computerized cognitive tests	Exercise, alcohol, depression, a history of COVID-19 infection	Loneliness was associated with worsening working memory executive function
Lee et al.^21^	Longitudinal/Korea	2,792	71.4 (NA)	NA	Voluntary stay-at-home	Social connection	Korean MMSE	Age, gender, educational attainment, employment status, and household income	Higher social connection was associated with higher cognitive score

Abbreviations: US, United States; UK, United Kingdom; W, White; B, Black; A, Asian; H, Hispanic; O, Other; SES, socioeconomic status; NA, Not available; MMSE-Brief, Mini-Mental State Examination-Brief; MMSE, Mini-Mental State Examination; BADL, basic activities of daily living; ICT, Information and communications technology; DM, diabetes; CVD, cardiovascular disease.

Note: *sample without dementia.

### Loneliness and cognition measurement methods

Measurements of loneliness varied significantly, ranging from psychometric scales such as the 3- and 16-item UCLA Loneliness Scale and the De Jong Gierveld Loneliness Scale^22^ to 1- to 2-item self-report questionnaires. Some studies also evaluated social isolation using the physical distancing and quarantine social isolation subscales of the Epidemic–Pandemic Impacts Inventory (EPII). Six of eight studies evaluated subjective cognitive or memory decline for cognitive assessment. In contrast, three studies utilized the objective measures of cognitive performance, employing computerized tests to assess attention, memory, and executive function, the Mini-Mental State Examination (MMSE)^20,21,23^.

### Relationship between loneliness and cognitive decline

Out of eight studies, six found a significant association between loneliness or social isolation and SCD during the COVID-19 pandemic in cognitively unimpaired older adults (Table 2)^14-21^, while only one study found a significant association between loneliness and SCD in executive function test^20^. One study linked lower social connectedness to lower global cognition during the pandemic^21^.

Noguchi et al.^14^ examined the relationship between social isolation and cognitive decline during the COVID-19 pandemic in Japan. During this period, the Japanese public health authorities recommended voluntary “stay-at-home” measures. The study included adults aged 70 years and older with no cognitive impairment at baseline. Cognitive function was self-reported using the Cognitive Performance Scale, assessed before and during the first year of the pandemic. Social isolation was evaluated based on the frequency of in-person meetings with friends, relatives, or neighbors through the question: “How often do you meet friends or relatives in person?” This question helped create a social isolation index for baseline and follow-up evaluations. Participants were categorized into four groups according to their social isolation status at baseline and follow-up: “remained non-isolated,” “isolated from non-isolation,” “non-isolated from isolation,” and “consistent isolation.” Throughout the follow-up period, the rate of participants who developed cognitive impairment was higher in the consistent isolation group (11.2%) compared to the non-isolation group (3.8%; p=0.0012). Multivariable logistic regression analysis indicated that the consistent isolation group had a statistically significantly higher risk of cognitive impairment than those who remained non-isolated (odds ratio [OR]=2.33, 95% confidence interval [CI]=1.07–5.05, p=0.033). Thus, individuals in the consistent isolation group experienced a notable increase in cognitive impairment risk at follow-up^14^.

Okely et al.^15^ investigated the connection between loneliness and cognitive decline in Scotland, particularly during the enforced stay-at-home measures implemented during the COVID-19 pandemic. Cognitive function was evaluated using a 5-item self-report questionnaire that assessed memory concerns, with scores ranging from 0 to 5; higher scores indicated more severe subjective memory issues. Loneliness was measured with a single question: “How much of the time during the past week have you felt lonely?” Participants rated their feelings on a scale of 1 to 4, with higher scores reflecting greater levels of loneliness. The study found no significant association between loneliness and changes in subjective memory decline scores before and during the pandemic (r=0.100)^15^.

Kobayashi et al.^16^ examined both between-person and within-person differences in loneliness and self-reported cognitive function during the COVID-19 pandemic in the United States, where social distancing measures varied, with some states implementing enforced and others voluntary stay-at-home requests based on the number of pandemic cases. A total of 2,204 individuals aged 55 years and older participated in the study. Loneliness was assessed using the 3-item UCLA Loneliness Scale^24^, with scores ranging from 3 to 9. Cognitive function was evaluated with the 6-item Patient-Reported Outcomes Measurement Information System (PROMIS), which captures negative thoughts about cognitive performance in daily life scenarios. The Cognitive Function scale was reverse-coded, so higher scores represented better cognitive outcomes. Loneliness and cognitive function assessments were conducted 4 months (August/September 2020), 6 months, 8 months, 10 months, and 12 months (April/May 2021) after the pandemic began. Between-person differences in loneliness scores were negatively correlated with cognitive function (β=−1.01 [-1.43, -0.59]), while within-person changes in loneliness also exhibited a negative association with cognitive function (β=−0.83 [-1.40, -0.26]). This indicates that individuals who felt more lonely than others had lower cognitive function. Additionally, those whose loneliness increased compared to their usual levels also reported a decline in cognitive function^16^.

Li et al.^17^ examined the role of information and communication technology (ICT) use in the relationship between loneliness, social isolation, and cognitive decline among community-dwelling older adults in Japan during voluntary “stay-at-home” measures aimed at mitigating the effects of the COVID-19 pandemic. The study targeted individuals aged 70–89 years. To assess cognitive decline, participants responded to a single question: “Do you feel rapid cognitive decline?” on a 4-point scale (strongly agree, slightly agree, slightly disagree, and strongly disagree). Based on their responses, the participants were categorized into two groups: the cognitive decline group (those who answered strongly agree or slightly agree) and the no cognitive decline group (slightly disagree or strongly disagree). Loneliness was measured using the Japanese version of the 3-item Loneliness Scale, while social isolation was assessed with a total score of six items. After controlling for covariates, loneliness (OR=1.30, 95%CI 1.01–1.69) and social isolation (OR 1.44, 95%CI 1.00–2.06) were significantly associated with SCD among participants aged 80 years and older. ICT use moderated this relationship. Interaction terms for ICT use with loneliness (OR 2.01, 95%CI 1.05–3.85) and social isolation (OR 2.45, 95%CI 1.05–5.75) significantly predicted SCD in the ≥80 years’ age group. Non-ICT users with high loneliness scores were more likely to experience SCD compared to ICT users with similar loneliness levels. Furthermore, older adults aged 80 years and above who utilized ICT did not appear to be affected by the negative impact of social isolation on cognitive decline^17^.

Nogueira et al.^18^ investigated the relationship between loneliness and cognitive decline in cognitively healthy older adults aged 50 years and older in Portugal during voluntary “stay-at-home” measures to control COVID-19 cases. Baseline assessments were conducted before and around 18 months after the pandemic started. The cognitive assessment included the MMSE, Montreal Cognitive Assessment (MoCA), Trail Making Test A/B, Digital Symbol Coding, Digit Span, and verbal fluency tasks. SCD was evaluated using the Cognitive Decline Complaints Scale (CDCS). Loneliness was assessed using the UCLA Loneliness Scale, consisting of 16 items. Notably, UCLA-16 scores statistically significantly correlated with CDCS scores (r=0.440; p<0.001) at 18 months. Despite worsened SCD, most objective cognitive tests revealed no significant differences in scores, and no correlations were found between social isolation or loneliness and objective cognitive decline^18^.

Durón-Reyes et al.^19^ established a connection between loneliness, social isolation, and cognitive function among older Mexican adults following enforced “stay-at-home” measures aimed at controlling COVID-19 cases. SCD was assessed using the Everyday Cognition Scale (E-Cog), which consists of 39 items evaluating an individual’s perception of their memory, language, visuospatial abilities, perceptual skills, and executive functioning. Higher scores on this scale indicate greater cognitive deterioration^23^. Loneliness was measured using the De Jong Gierveld Loneliness Scale, where higher scores reflect increased feelings of loneliness. At the same time, social isolation was evaluated using the Physical Distancing and Quarantine subscale of the EPII. A higher score suggests higher social isolation. Loneliness scale scores (rho=0.219, p<0.005) and EPII Physical Distancing and Quarantine subscale scores (rho=0.178, p≤0.05) correlated with E-Cog scores^19^.

Corbett et al.^20^ utilized longitudinal data from the PROTECT study to evaluate the impact of the pandemic on cognition in adults aged 50 years and older in the UK during the enforced “stay-at-home” measures. Computerized neuropsychological data, encompassing executive function, working memory, and attention, were collected from the same participants before the COVID-19 pandemic (March 1, 2019–February 29, 2020) and during its first (March 1, 2020–February 28, 2021) and second years (March 1, 2021–February 28, 2022). Loneliness was assessed using two items from a broader mental health questionnaire, asking participants, “Do you often feel alone?” and “How often, in the past week, did you feel alone?” The findings indicated that loneliness was associated with lower executive function in the second year of the pandemic (β=-1.253; 95%CI -1.951 to -0.555; p=0.006) in the whole cohorts, which included individuals who had acquired COVID-19 infection and those who had developed mild cognitive impairment (MCI) during the pandemic^20^.

Lee et al.^21^ estimated the causal effect of social connectedness on cognitive functions among older Korean adults during a voluntary “stay-at-home” order implemented to control the pandemic. The study utilized data from the Korean Longitudinal Study of Aging, a nationally representative longitudinal survey of non-institutionalized Koreans aged 45 years and older. Social connectedness was measured based on the frequency of meetings with friends, relatives, or neighbors, while cognitive function was assessed using the Korean version of the MMSE. Social distancing measures during the COVID-19 period led to a statistically significant decrease in social interactions, with the frequency of meeting friends, relatives, and neighbors declining from the pre-COVID-19 (mean=5.54) to post-COVID-19 (mean=4.54; p<0.0001) period. An average one-unit increase in participants’ frequency of meeting family and friends was associated with higher cognitive scores on the Korean MMSE (random effect: 0.147; p<0.01; fixed effect: 0.503; p<0.05). This indicates that increased social interaction correlates with improved cognitive scores. The social distancing measures enacted due to the COVID-19 pandemic may have influenced various factors, subsequently affecting cognitive function.

## DISCUSSION

This study synthesizes evidence indicating that experiences of loneliness and social isolation were associated with SCD in cognitively unimpaired older adults during the COVID-19 pandemic. Notably, only one study connected loneliness and objective cognitive decline in the executive function domain. These findings suggest that loneliness resulting from social distancing measures, whether voluntary or enforced, is linked to an increased perception of cognitive decline among non-institutionalized cognitively unimpaired older adults.

Loneliness can manifest as a profound sense of disconnection from social circles, which older adults often experience due to various life changes, such as losing friends or family members^1,2,7^. In this review, multiple studies have identified an association between loneliness and SCD^14-21^, supporting findings from previous research^25-29^. For instance, evidence indicates that older adults reporting higher levels of loneliness are at an elevated risk for developing dementia and other cognitive disorders^26-29^.

The global implementation of social distancing measures during the COVID-19 pandemic resulted in abrupt changes to daily routines and significantly reduced opportunities for social engagement, leading many individuals to feel isolated and unsupported^8,25,28,30^. This situation intensified feelings of loneliness and its accompanying psychological consequences^8,28^. Whether due to government-imposed lockdowns or voluntary isolation, the overall result was a notable increase in loneliness among older adults.^8,26-30^ This shared experience of diminished social opportunities fostered a collective sense of isolation, particularly as many older adults struggled adapting to virtual forms of communication, further exacerbating their feelings of loneliness and disconnection^9,25-28^.

Noguchi et al.^14^ demonstrated that individuals experiencing consistent isolation exhibited a significant increase in the risk of cognitive impairment at a 6-month follow-up during the first year of the pandemic. Notably, Noguchi et al. utilized self-reported cognitive function to classify MCI^14^. Similarly, Li et al.^17^ found a significant association between social isolation and SCD among participants aged 80 years and older. However, this association was not observed in younger adults. Interestingly, using technology to maintain communication with family, friends, and relatives moderated this relationship. In contrast, Durón-Reyes et al.^19^ observed a similar association during the COVID-19 pandemic among older Mexican adults, regardless of age.

Among the selected studies, three employed objective cognitive measures to assess cognitive changes before and after the pandemic^20,21^. Nogueira et al.^18^ found a decrease in Trail Making Test (TMT) and Digit Symbol Coding (DSC) scores. Lee et al.^21^ reported a small but significant decline in MMSE scores before and after the COVID-19 pandemic. Corbett et al.^20^ found considerable worsening in executive function and working memory in the first and second years of the pandemic across the entire cohort. Only Corbett et al.^20^ found a significant association between loneliness and cognitive function. Particularly, higher loneliness was associated with executive function in the second year of the pandemic in a sample of cognitively normal older adults and participants who had acquired COVID-19 infection and those who had developed MCI after the pandemic^18^. Conversely, Lee et al.^21^ observed that higher levels of social connection correlated with better global cognition.

Our evidence synthesis indicates that loneliness and social isolation during the COVID-19 pandemic were consistently linked to SCD in non-institutionalized older adults without dementia. These findings support previous reviews^25,28^, which suggest that loneliness and social isolation during the pandemic may exacerbate cognitive deterioration due to stress and changes in neurobiological mechanisms, such as increased cortisol secretion and its detrimental effects on brain health. However, our study found that the relationship between loneliness and cognitive decline in individuals without cognitive impairment was not observed when objective measures of cognitive function were used, as assessed by neuropsychological tests instead of self-perceived cognitive performance. This observation raises the question of whether the perceived cognitive decline reported by older adults truly reflects cognitive deterioration or is influenced by mood states. It is plausible that the feelings of loneliness and social isolation experienced during the pandemic led to increased anxiety, depression, and stress, ultimately affecting individuals’ self-assessments of cognitive function. Several studies conducted outside the pandemic context have identified a strong relationship between depression and SCD, and both are associated with an increased risk of ADRDs. However, it remains unclear whether they reflect independent neurobiological mechanisms^31-33^. Undoubtedly, depression is a factor to consider while analyzing the relationship between loneliness and cognitive decline. Among the studies included in our review, half incorporated depression, anxiety, and mood as covariates in their analytical models^33^. Previous reviews discussed various factors influencing loneliness, social isolation, and cognitive outcomes, including sex differences, socioeconomic status, and mental health^8,25,28^. Therefore, whether the connection between loneliness and SCD during the pandemic is an indication of the potential dementia risk remains unclear. Future studies examining the relationship between loneliness and cognitive decline during the pandemic should consider the analysis of ADRD biomarkers and measures of objective cognitive decline while controlling for mental health covariates.

In addition, our findings emphasize the critical role of social connections in maintaining cognitive health and reveal potential cognitive risks associated with loneliness and prolonged social isolation, even among cognitively healthy older adults. In a study of 2,792 older adults in Korea, Lee et al.^21^ demonstrated that an increase in social connectedness during the pandemic — specifically, more frequent meetings with friends, relatives, or neighbors — was associated with higher MMSE cognitive scores. Similarly, Li et al.^17^ demonstrated that ICT use (i.e., sending e-mail, browsing websites, using LINE, Skype, and ZOOM to communicate, and accessing social networking media) moderated the relationship between loneliness, social isolation, and cognition in older adults during the pandemic. Non-ICT users with high loneliness and social isolation scores were more likely to experience SCD compared to ICT users with high loneliness scores^17^. This evidence suggests that the pandemic adversely affected social connectedness in older adult populations, which consequently might have impacted cognition. These results are consistent with broader global observations, highlighting an increase in loneliness linked to public health measures that restricted social gatherings and enforced isolation during the pandemic^5,8,34^. Social engagement is vital for maintaining cognitive health^17,21^. Positive interactions and stimulating conversations can enhance cognitive reserve, empowering older adults to reduce their risk of cognitive disorders^2,3,17,21^. Consequently, as social opportunities diminish, the chances for mental stimulation also decrease, potentially accelerating cognitive decline. Therefore, extended periods of social isolation and loneliness during the pandemic may have exacerbated ongoing neurodegenerative processes, leading to harmful effects on cognitive function. Continuous monitoring of loneliness and isolation levels is crucial, even post-pandemic, due to their potential negative impact on aging trajectories and quality of life in this vulnerable population.

Our systematic review revealed that loneliness and social isolation are consistently associated with worsening SCD during the COVID-19 pandemic. This offers unique insights into how prolonged loneliness may negatively impact cognitive trajectories. However, important questions remain regarding the persistence and clinical significance of the observed cognitive declines. Longitudinal research is essential to determine whether changes in cognitive performance related to loneliness persist over time or whether cognition improves with the resumption of social interactions. Moreover, examining the biomarkers of pre-clinical ADRDs can also deepen our understanding of this unprecedented social disruption in late-life cognitive health.

This review builds on prior evidence^5,28,34^ by concentrating on community-dwelling older adults without cognitive impairment. Additionally, we clarify the relationship between loneliness and cognitive function, demonstrating a strong association between loneliness and SCD but not necessarily with objective cognitive decline. However, substantial research gaps remain. The limited number of published studies restricts our conclusions, and the variability in the definitions of loneliness and cognitive measures used limits an in-depth analysis through a meta-analysis approach.

Ongoing research into modifiable factors such as loneliness is crucial to understanding the long-term cognitive consequences and developing strategies to enhance cognitive resilience in older adults amidst the ongoing effects of the pandemic.

In conclusion, this review demonstrated that prolonged loneliness due to social distancing and isolation was consistently linked to SCD in non-institutionalized older adults without dementia. However, it remains uncertain whether loneliness associated with the COVID-19 pandemic also contributed to objective cognitive decline. The pandemic has highlighted an ongoing issue: the long-term, indirect effects of social isolation on cognition and mental health. Older adults may continue to face an increased risk of cognitive decline and persistent loneliness, even as pandemic restrictions ease. Understanding the lingering impacts of extended social disruption will aid in identifying individuals most vulnerable to ongoing effects on brain health and well-being.

## Data Availability

The datasets generated and/or analyzed during the current study are available from the corresponding author upon reasonable request.
